# *CCN3, POSTN, and PTHLH* as potential key regulators of genomic integrity and cellular survival in iPSCs

**DOI:** 10.3389/fmolb.2024.1342011

**Published:** 2024-02-05

**Authors:** Nuha T. Swaidan, Nada H. Soliman, Ahmed T. Aboughalia, Toqa Darwish, Ruba O. Almeshal, Azhar A. Al-Khulaifi, Rowaida Z. Taha, Rania Alanany, Ahmed Y. Hussein, Salam Salloum-Asfar, Sara A. Abdulla, Abdallah M. Abdallah, Mohamed M. Emara

**Affiliations:** ^1^ Basic Medical Sciences Department, College of Medicine, QU Health, Qatar University, Doha, Qatar; ^2^ Neurological Disorders Research Center, Qatar Biomedical Research Institute, Hamad Bin Khalifa University, Doha, Qatar; ^3^ College of Health and Life Sciences, Hamad Bin Khalifa University, Doha, Qatar; ^4^ School of Medicine, New Giza University, Giza, Egypt

**Keywords:** iPSCs, ESCs, transcription factors, genomic integrity, cellular survival

## Abstract

Reprogramming human somatic cells into a pluripotent state, achieved through the activation of well-defined transcriptional factors known as OSKM factors, offers significant potential for regenerative medicine. While OSKM factors are a robust reprogramming method, efficiency remains a challenge, with only a fraction of cells undergoing successful reprogramming. To address this, we explored genes related to genomic integrity and cellular survival, focusing on iPSCs (A53T-PD1) that displayed enhanced colony stability. Our investigation had revealed three candidate genes *CCN3*, *POSTN*, and *PTHLH* that exhibited differential expression levels and potential roles in iPSC stability. Subsequent analyses identified various protein interactions for these candidate genes. *POSTN*, significantly upregulated in A53T-PD1 iPSC line, showed interactions with extracellular matrix components and potential involvement in Wnt signaling. *CCN3*, also highly upregulated, demonstrated interactions with TP53, CDKN1A, and factors related to apoptosis and proliferation. *PTHLH*, while upregulated, exhibited interactions with CDK2 and genes involved in cell cycle regulation. RT-qPCR validation confirmed elevated *CCN3* and *PTHLH* expression in A53T-PD1 iPSCs, aligning with RNA-seq findings. These genes’ roles in preserving pluripotency and cellular stability require further exploration. In conclusion, we identified *CCN3*, *POSTN*, and *PTHLH* as potential contributors to genomic integrity and pluripotency maintenance in iPSCs. Their roles in DNA repair, apoptosis evasion, and signaling pathways could offer valuable insights for enhancing reprogramming efficiency and sustaining pluripotency. Further investigations are essential to unravel the mechanisms underlying their actions.

## 1 Introduction

The reprogramming of human somatic cells into a pluripotent state has been accomplished by the ectopic expression of well-defined transcriptional factors such as *OCT4* (also known as *POU5F1*), *SOX2*, *KLF4*, *MYC* (also known as c-MYC); these factors are collectively referred to as the OSKM factors (Yamanaka, 2012; Swaidan et al., 2020). Human induced pluripotent stem cells (hiPSCs), which possess embryonic stem cell (ESC)-like characteristics, have increased the possibility of generating patient-specific cells (Dimos et al., 2008; Soldner et al., 2009). These hiPSCs are distinguished by their ability to self-renew and differentiate into any cell type of the body. Consequently, these cells can serve purposes such as comprehending disease progression, developing and testing novel drugs, and conducting autologous replacement therapies for degenerated cells (Dimos et al., 2008). This is particularly relevant to degenerative diseases of the central nervous system (CNS) such as Parkinson’s disease (PD), in which dopaminergic neurons are lost (Soldner et al., 2009).

PD is the second most common chronic progressive neurodegenerative disease after Alzheimer’s disease. It is characterized by the loss of the nigrostriatal dopamine-releasing neurons. Despite the fact that genetic factors are implicated in the pathogenesis of PD, the majority of PD cases are sporadic, not associated with a known genetic mutation (Soldner et al., 2009). The pathological hallmark of PD is the aggregation and deposition of abnormal α-synuclein protein within the neuronal cell bodies (Stefanis, 2012). Numerous mutations have been reported in the gene responsible for encoding this protein. The α-synuclein point mutation (A53T) is believed to be the most frequent and associated with the familial type of PD (Swaidan et al., 2020).

While OSKM factors have demonstrated a robust method for reprogramming somatic cells that has potential for regenerative medicine, the process is still inefficient since only a small fraction of cells undergoes a complete and successful reprogramming (Swaidan et al., 2020). To overcome low reprogramming efficiency, several studies have examined factors that could enhance the activation of the pluripotency pathway and prevent the differentiation of stem cells (M. Wang et al., 2017; Bogliotti et al., 2018; Xu et al., 2016; Theka et al., 2017). In our earlier research, we pinpointed five genes (*GBX2, NANONGP8, SP8, PEG3, and ZIC1*) exhibiting differential expression in iPSCs generated from fibroblast cells. These genes maintained their differential expression regardless of the presence or absence of the PD A53T mutation, while also showcasing interactions with OSKM factors (Swaidan et al., 2020). Building upon our prior research, this paper focuses on a distinct sample (named A53T-PD1) that displayed enhanced colony integrity and stability in comparison to other samples (A53T-PD2, ID-PD and HC). This observation holds true despite the fact that a smaller number of colonies were formed during the reprogramming process of this sample.

Hence, with the aim of enhancing the regeneration process’s efficiency, our focus lies in investigating genes related to genomic integrity/stability and cellular survival. These genes exhibit differential expression in A53T-PD1 cells and could potentially be genetically modified or suppressed to bolster and sustain iPSC pluripotency.

## 2 Materials and methods

### 2.1 Cell lines and cell culture

In this study, four established human iPS cell lines (HC, ID-PD, A53T-PD1, and A53T-PD2) were employed (*n* = 3 per iPS cell line, total = 12 samples). These stem cells were grown using a feeder-free system with Matrigel (Corning) for cell adherence and maintained in StemFlex media (Termo Fisher Scientifc) under controlled conditions at 37°C and 5% CO_2_ incubators. Cells were fed every other day, and passage was performed when cell confluency reached approximately 80%, typically within four to 5 days after the previous passage. The passage process involved pre-coating of 35 mm dishes with Matrigel for 30 min, 2 h, or overnight, washing colonies with 1 mL Dulbecco’s Phosphate-Buffered Saline (DPBS), gently dissociating the cells into appropriately sized colonies (∼100 µm) using 500 µL of non-enzymatic reagent (ReLeSR; StemCell Technologies), collecting these colonies with StemFlex media, centrifuging them for 4 min at 800 RPM and 22°C, resuspending the resulting pellet in 1 mL fresh StemFlex media, and finally evenly distributing 70 μL of cells onto the freshly prepared dishes containing 2 mL fresh StemFlex media.

### 2.2 RNA and RT-qPCR

Total RNA was extracted with a Direct-zol RNA MicroPrep Extraction kit (Zymo Research) according to manufacturer’s instructions. Then, cDNA was synthesized using Superscript IV, First-Strand Synthesis System kit (Invitrogen). The RT-qPCR technique was performed using TaqMan Fast Advanced Master Mix (Applied Biosystems) and TaqMan probes (20×) according to the manufacturer’s protocol. The list of genes: Assay name: POSTN, Assay ID: Hs0166750, PTHLH, Assay ID: Hs00174969, NOV (CCN3), Assay ID: Hs00159631 and housekeeping gene GAPDH, Assay ID: Hs02786624. The total reaction volume was 10 µL per well. RT-qPCR reaction was run in triplicates using Applied Biosystems 7500 Fast Dx Real-Time PCR Instrument under default conditions (95°C for 10 min and then PCR reaction: 40 cycles of 95°C, 15 s and 60°C, 1 min).

### 2.3 RNA sequencing (RNA-Seq)

The transcriptomic sequencing process encompassed two key steps: the first step involved the preparation of mRNA libraries, followed by RNA sequencing. Initially, the quality of the extracted total RNA samples was assessed using a 2100 Bioanalyzer equipped with the Agilent RNA Nano 6000 chip. Subsequently, we carried out mRNA library preparation using the TruSeq Stranded mRNA Sample Prep LS kit (Illumina). To ensure the quality and quantity of the generated mRNA libraries, a thorough quality control analysis was conducted using the Agilent DNA 1000 chip on a 2100 Bioanalyzer, along with the quantification of DNA library templates using a Qubit assay. For cluster generation, indexed DNA libraries were normalized to 10 nM and placed in a diluted cluster template (DCT) plate, after which they were combined in equal volumes in a pooled DCT plate (PDP). The final step involved RNA sequencing, which was performed utilizing the HiSeq 4000 system (Illumina) at the QBRI genomic core facility.

### 2.4 RNA-seq analysis

We employed the Bcl2fastq Conversion Software to perform two crucial tasks: firstly, to demultiplex the data, and secondly, to convert the BCL files generated by Illumina sequencing systems into standard FASTQ file formats, which are essential for subsequent RNA-Seq analysis. Quality control checks were conducted using FastQC, a specialized tool designed for high-throughput sequence data analysis, accessible at (https://www.bioinformatics.babraham.ac.uk/projects/fastqc/).

The subsequent analysis followed the standard QIAGEN bioinformatics CLC Genomics Workbench 20.0 pipeline (https://digitalinsights.qiagen.com). This sequential pipeline comprised four key stages: 1) pre-processing of raw sequencing reads, 2) mapping of reads to a reference genome, 3) quantification of genes and transcripts, and 4) differential expression analysis. Data normalization was performed relative to the healthy control iPS cell line, which served as the baseline for comparison.

### 2.5 Gene ontology molecular analysis

We utilized the Protein Analysis Through Evolutionary Relationships (PANTHER) 14.1 platform, accessible at (http://www.pantherdb.org), as an online classification system. PANTHER is specifically designed for the identification and categorization of proteins and their corresponding genes based on their family/subfamily, molecular function, biological processes, and pathways in which they play integral roles within the cellular context. This software served as a valuable tool for filtering and pinpointing genes relevant to our areas of focus, which encompassed cellular integrity and genomic stability.

### 2.6 Protein-protein interaction analysis

We employed the Search Tools for the Retrieval of Interacting Genes (STRING) 10.5, an online software dedicated to the exploration of gene and protein interactions, accessible at (https://string-db.org/). Our primary objective with STRING was to establish predicted interactions between our selected genes of interest and protein-encoding genes that are involved in cell cycle checkpoints, DNA repair and replication, *TP53* phosphorylation, and apoptosis inhibition. Through the STRING platform, we did not only visualize the nature of interactions between these proteins but also examined the underlying evidence supporting these interactions.

### 2.7 Databases and literature review

A comprehensive literature review was conducted using medical search engines such as PubMed, Medline, UpToDate, Scopus, Access Medicine, Genetic Home Reference, and Access Genetics. Specific keywords were employed such as “the name of the chosen candidate gene,” “pluripotency/pluripotent,” “reprogramming,” “genomic stability/integrity in Embryonic stem cells/ESCs,” and “induced pluripotent stem cells/iPSCs”. Relevant articles were screened and their findings documented for confirmation of transcriptional factors’ involvement in genomic integrity and cellular survival.

### 2.8 Statistical analysis

Statistical analyses were conducted using GraphPad Prism 8.4.3 (GraphPad Software, Inc., San Diego, CA). Data were presented as the mean ± standard deviation (SD) for each group (N = 3). Statistical significance was assessed through the Mann–Whitney *U* test and defined as *p*-values less than 0.05 (*p* < 0.05) or 0.01 (*p* < 0.01).

## 3 Results

### 3.1 A set of genes involved in molecular function regulation, including DNA binding, biological adhesion and intracellular signalling

**FIGURE 1:**
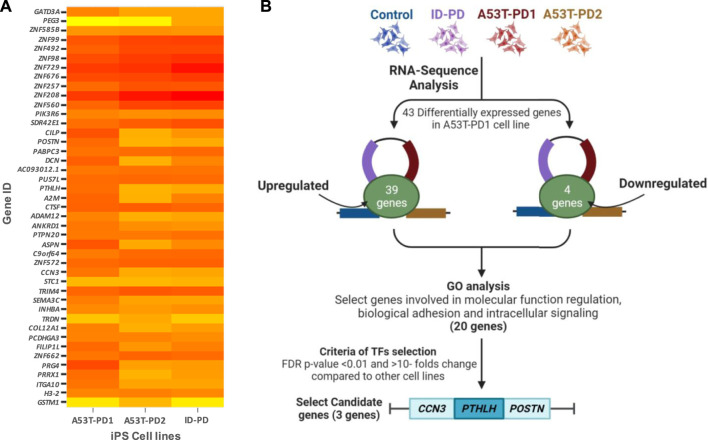
Comprehensive visualization of study findings. **(A)** Heatmap of differentially expressed genes in A53T-PD1 cell line. **(B)** Illustrative flowchart summarizing the procedural pathway that led to the identification of three candidate genes within the A53T-PD1 iPS cell line as potential contributors to genomic integrity and pluripotency maintenance in iPSCs.

Due to the superior colony stability observed in A53T-PD1 iPSCs, we turned our focus to the identification of differentially expressed candidate genes across distinct iPS cell lines. Utilizing RNA sequencing data, we conducted a comparative analysis of differentially expressed genes between A53T-PD1 and other iPS cell lines: HC, ID-PD, and A53T-PD2. Our selection criteria involved genes exhibiting a false discovery rate (FDR) of less than 0.05 specifically in A53T-PD1 cell line. This screening resulted in the identification of a total of 43 genes, comprising 39 genes with upregulated expression and 4 genes displaying downregulated expression (Figure 1).

**FIGURE 2:**
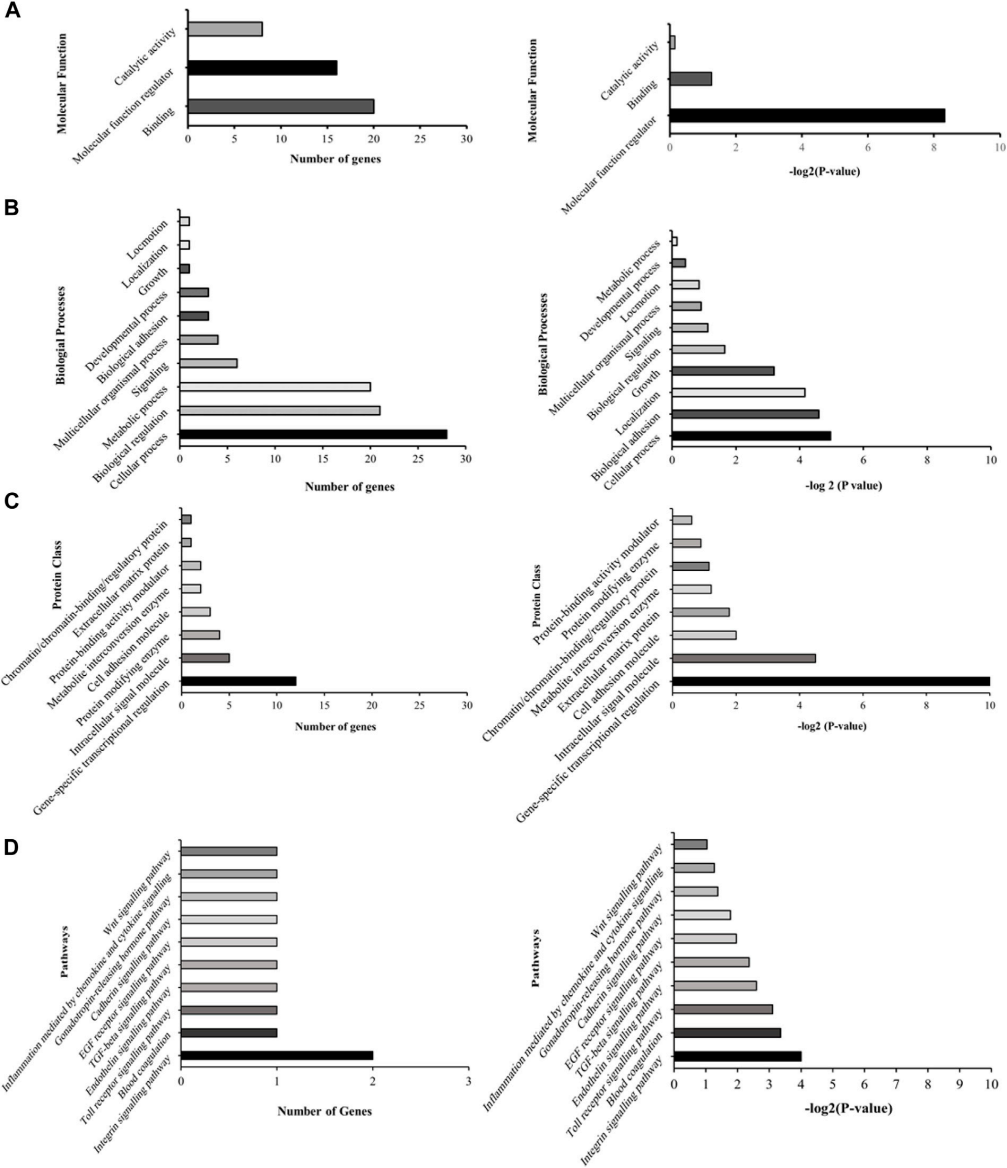
Illustrative graphs for gene ontology (GO) analysis. The left panels **(A–D)** display representative graphs categorizing the 43 differentially expressed genes based on their cellular roles and functions. The right panels **(A–D)** demonstrate the statistical significance of these categorized genes.

Subsequent to this discovery phase, we proceeded to subject the 43 genes to Gene Ontology analysis (GO), in order to systematically classify them based on their roles and functions within the cellular context (Figure 2). Our selection process was grounded in the prioritization of cellular processes that bear utmost relevance to both cellular integrity and genomic stability. These encompassed processes such as molecular function regulation, DNA binding, biological and cell adhesion, as well as intracellular signaling. Notably, while the statistical significance threshold was not met for DNA binding and cell adhesion molecules (with FDR *p*-values of 0.42 and 0.25, respectively), the attributes of molecular function regulation, biological adhesion, and intracellular signaling exhibited statistical significance (FDR *p*-values of 0.003, 0.04, and 0.04, respectively). Guided by these findings, our attention was directed towards refining the gene selection criteria to those involved in molecular functions, biological processes, and protein classes, with a focus on genes that are implicated in molecular function regulation, biological adhesion, and intracellular signaling (as outlined in Figure 2).

### 3.2 Selection of candidate genes in A53T-PD1 cell line

We successfully identified a total of 20 genes, effectively categorized into two distinct groups. The initial group comprised 11 zinc finger transcription regulator genes, integral to the orchestration of molecular function regulation. The remaining 9 genes, namely, *CCN3, INHBA, PTHLH, PIK3R6, PEG3, H3.2, POSTN, PCDHGA3,* and *STC1*, demonstrated their involvement across various functional domains. Further delving into the expression patterns of these 20 genes within each iPS cell line occurred through an in-depth analysis, utilizing the differential expression data generated in relation to control iPSCs.

**FIGURE 3:**
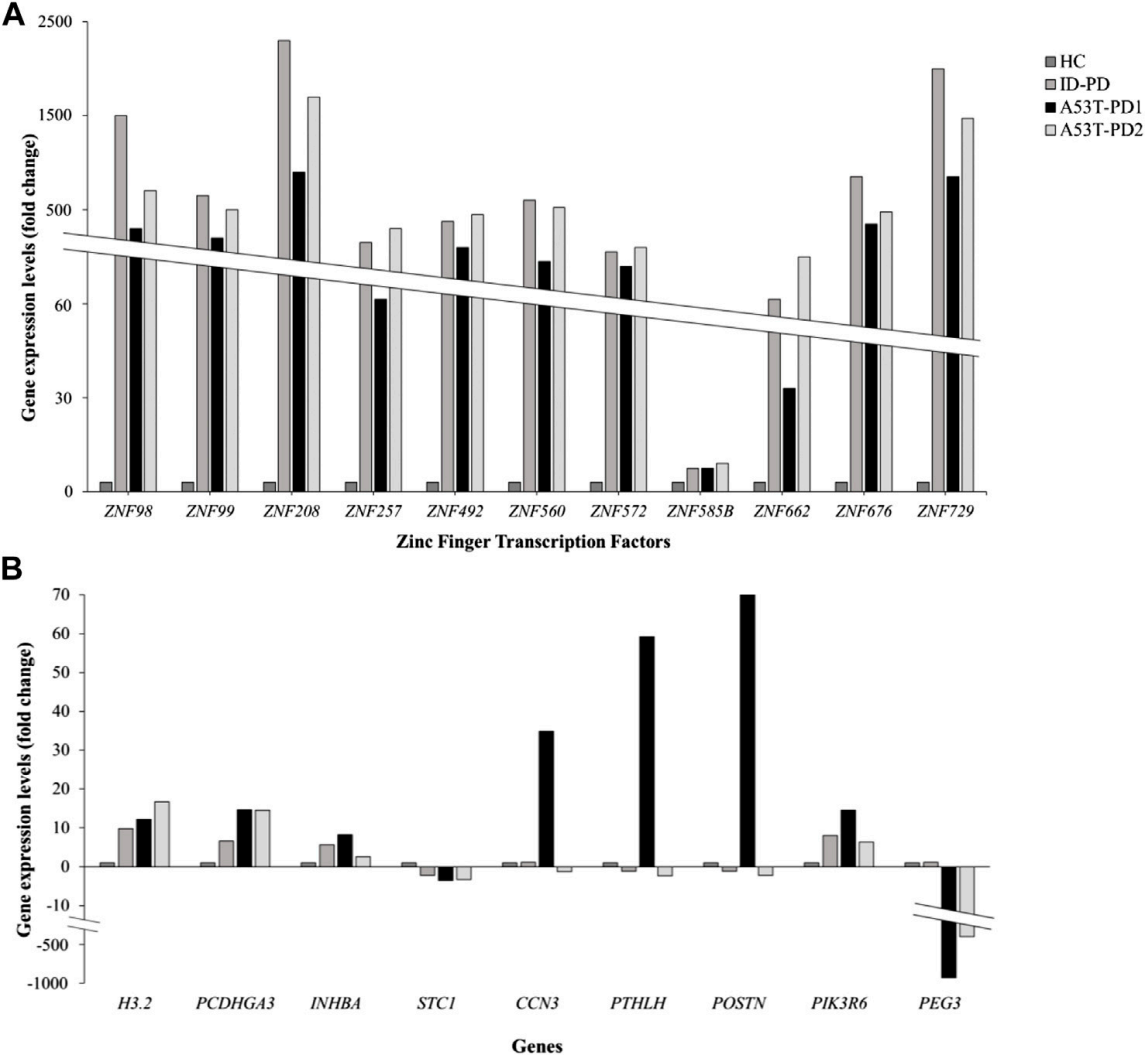
Expression profiling of 20 genes involved in genomic integrity and cellular survival. Gene expression levels for a panel of 20 genes associated with genomic integrity/stability and cellular survival were analyzed using RNA-Seq data and represented in two separate graphs. **(A)** Illustrates the expression levels of 11 zinc finger transcription factors expressed in different iPS cell lines. **(B)** Illustrates the expression levels of the remaining 9 genes expressed in iPS cell lines.

For the zinc finger transcription regulator genes, a consistent and statistically significant upregulation was observed across all cell lines (FDR <0.01), compared to control iPSCs (illustrated in Figure 3A). As a result of this uniformity, these genes were subsequently excluded from further gene selection. Conversely, among the remaining genes (Figure 3B), the highest upregulation in A53T-PD1 cell line was notably exhibited by *POSTN*, soaring 70-fold (FDR <0.001), followed closely by *PTHLH* with a 59-fold increase (FDR <0.001). In contrast, both *POSTN* and *PTHLH* showed downregulation in ID-PD and A53T-PD2 cell lines. Additionally, *CCN3*, *INHBA*, and *PIK3R6* displayed substantial upregulation in A53T-PD1 cell line, significantly exceeding other cell lines (FDR <0.001 for all).

H3.2 displayed an upregulation of 12-fold in A53-PD1 cell line, in contrast to 16-fold in A53T-PD2 and 9-fold in ID-PD cell lines, all with an FDR <0.001. *PCDHGA3*, on the other hand, exhibited consistent upregulation across all cell lines, demonstrating a similar 15-fold change in both A53T-PD1 and A53T-PD2 cell lines (FDR <0.001). Unlike all the previously mentioned genes, *STC1* and *PEG3* displayed downregulation. While *STC1* demonstrated downregulation across all cell lines without significant variation among them, *PEG3* showcased its most pronounced downregulation in A53T-PD1 cell line at a staggering −990-fold change (FDR <0.001), succeeded by A53T-PD2 cell line at −443-fold change (FDR <0.001) (Figure 3B).

In the quest for a final selection of candidate genes potentially contributing to the generation of the most stable iPSC colonies in A53T-PD1 cell line, we employed two stringent criteria: (a) FDR *p*-value <0.01 and (b) a significant upregulation or downregulation in A53T-PD1 cell line, displaying a variation of more than 10-fold compared to other cell lines. Consequently, *INHBA*, *PIK3R6*, *H3.2*, *STC1*, and *PCDHGA3* were disregarded. It is important to highlight that *PEG3* was excluded from consideration, as it had already been analyzed in our previous work (Swaidan et al., 2020). Therefore, this rigorous process left us with a final selection of three genes: *CCN3*, *POSTN*, and *PTHLH*.

### 3.3 Protein-protein interaction analysis

Following gene selection, we utilized STRING software to predict potential protein interactions associated with our chosen candidate genes. Individually, each gene underwent scrutiny to anticipate its feasible associations with other proteins. Additionally, each gene underwent evaluation in conjunction with a cluster of genes linked to cell cycle regulation and DNA repair/replication.

**FIGURE 4:**
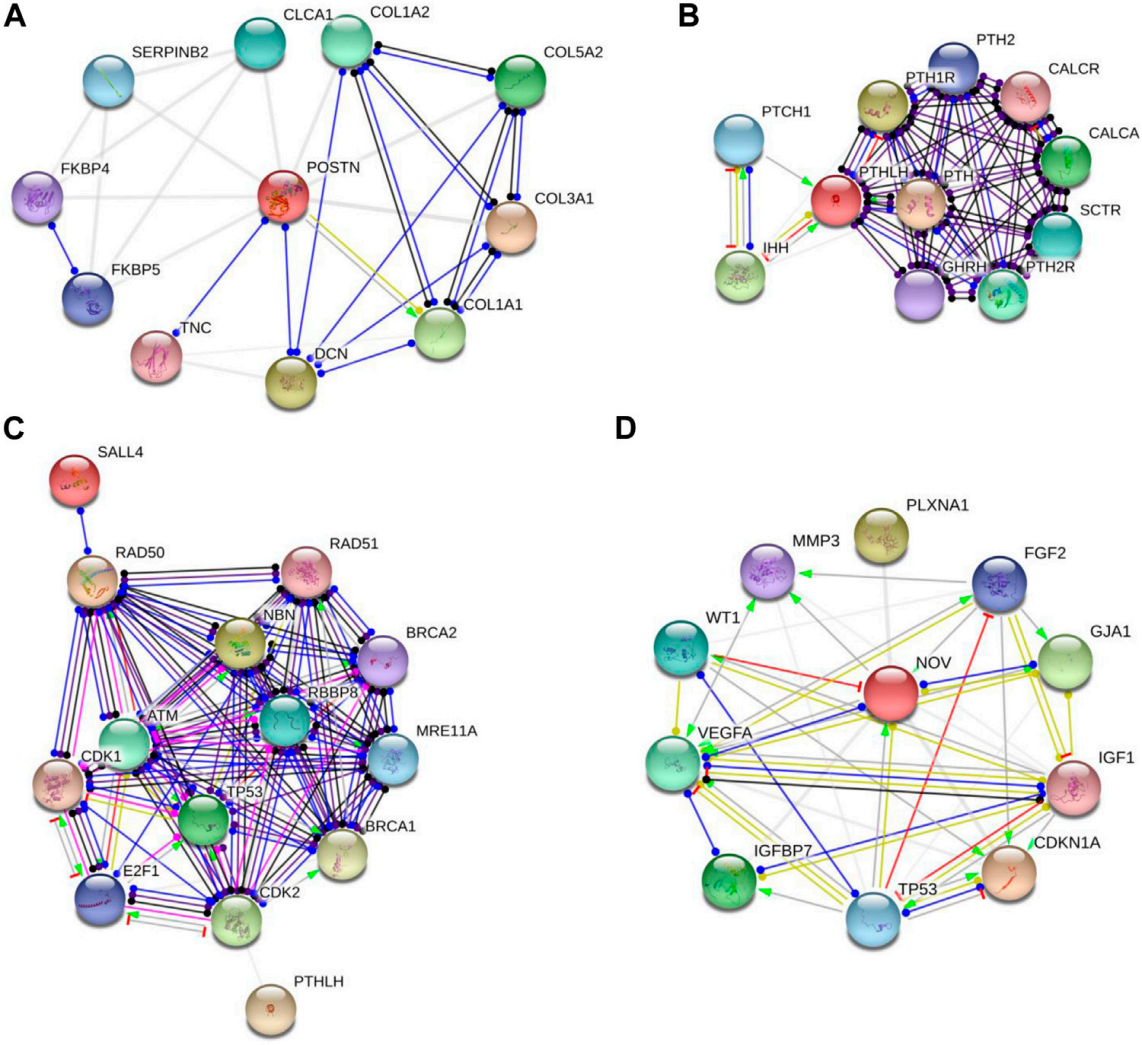
Protein-protein interaction networks revealed by String software analysis. **(A,B and D)** Protein interaction network involving POSTN, PTHLH and CCN3 (also known as NOV). **(C)** Predicted interaction network of genes responsible for preserving cellular survival and genomic integrity in ESCs. Each network node represents all the proteins generated by a single protein-coding gene locus. Colored nodes denote query proteins and first shell of interactors, while white nodes indicate second shell of interactors. Empty nodes signify proteins with unknown 3D structures, while filled nodes denote proteins with known or predicted 3D structures. It's important to note that these protein associations, while significant, do not necessarily indicate physical binding. The network edges in this figure represent molecular interactions between the associated proteins, with line shape indicating the predicted mode of action. Line colors convey different action types, such as binding (blue line), activation (green line), inhibition (red line), reaction (black line), catalysis (purple line), transcriptional regulation (yellow line), and posttranslational modification (pink line). Unidentified interactions are represented by grey lines. Arrowed lines indicate a positive effect, bar-headed lines signify a negative effect, and circular-ended lines denote an unspecified effect.

An illustrative instance of this predictive analysis is evident in *POSTN*, which exhibited a remarkable 70-fold upregulation in A53T-PD1 cell line. This gene was predicted to stimulate *COL1A1* expression, contributing to the synthesis of a substantial portion of type 1 collagen (as indicated in Figure 4A through the green arrow). Furthermore, *POSTN* demonstrated unknown interactions with other collagen-related genes, including *COL1A2*, *COL5A2*, and *COL3A1*, as well as the *DCN* gene, responsible for encoding the decorin protein. Decorin, in turn, contributes to extracellular matrix strength and cell stability. Notably, *POSTN* also exhibited a binding interaction with *TNC*, which encodes Tenascin C, a crucial extracellular matrix protein involved in guiding migrating neurons and axons during development, synaptic plasticity, and neuronal regeneration (as indicated in Figure 4A by the blue line).

In our subsequent exploration, we aimed to determine whether *POSTN* had any potential interactions with gene sets pivotal to fundamental cellular processes such as cellular stability and genomic integrity in ESCs. These encompassed genes that are responsible for DNA repair and replication, phosphorylation of p53, and other molecules like Rb and ATM. For this analysis, we included genes related to cell cycle checkpoints (*CDK1/2*), the MRN complex intrinsic to the homologous recombination pathway of DNA repair (*MRE11*, *RAD50*, and *NBS1*), alongside *BRCA1/2*, *RAD51*, and *SALL4*. However, it's important to highlight that *POSTN* did not display any predicted interactions with the aforementioned genes.

Similarly, *PTHLH* which experienced a significant upregulation in A53T-PD1 cell line (an impressive 59-fold increase) was predicted to exert an inhibitory influence on the *IHH* gene (as indicated in Figure 4B by the red line). This particular gene plays a pivotal role in governing the differentiation of progenitor cells into osteoblasts, thus exerting a significant influence over cellular differentiation dynamics. Additionally, *PTHLH* exhibited interactions with parathyroid hormone-related genes including *PTH1R*, *PTH2*, *PTH2R*, as well as calcitonin-related genes such as *CALCR* and *CALCA*. Upon closer examination of *PTHLH’s* interactions with the aforementioned gene set that is vital to substantial cellular processes, an intriguing and as yet unidentified interaction emerged with cyclin-dependent kinase 2 (*CDK2*) (Figure 4C). *CDK2*, a pivotal player in cell cycle control, undertakes the phosphorylation of *TP53*, thereby impeding apoptosis. Furthermore, *CDK2’s* involvement extends to the activation of *E2F1* gene expression, an essential precursor for the initiation of DNA synthesis. The orchestrated actions of *CDK2* and *E2F1* contribute to the hyperphosphorylation of the Rb protein-encoding gene, ultimately facilitating ESCs’ efficient transition into the S phase of the cell cycle and thereby shortening the duration of the G1 phase.

Interestingly, further exploration of *CCN3* (which exhibited a significant upregulation, 34.9-fold increase in A53T-PD1 cell line) revealed that *CCN3’s* transcriptional regulation, as an extracellular adhesion-related protein, is directly influenced by the *TP53* gene that is recognized as a pivotal tumor suppressor gene. This gene wields substantial influence over cellular survival and apoptotic processes through the p53 signaling pathway (Figure 4D).

Moreover, *CCN3* exhibited a mysterious interaction with *CDKN1A*, a crucial player within the p53 pathway responsible for orchestrating cell apoptosis and survival. Notably, *CDKN1A* serves as an essential mediator, enabling p53/TP53 to execute its role as a proliferation inhibitor in response to DNA damage. Furthermore, *CCN3’s* interactions extended to *VEGFA*, where it contributed to elevated expression levels and binding activity, while concurrently exerting inhibitory effects on *WT1*, leading to decreased expression levels. This latter gene, *WT1*, assumes the role of suppressing cellular apoptosis. Additionally, *CCN3’s* involvement encompassed the transcriptional regulation of the *IGF1* gene, known for its capacity to hinder cellular apoptosis by activating the PI3K/AKT pathway and inciting cell proliferation. This effect stands in contrast to the apoptotic influence of p53, thus creating a complex interplay (illustrated in Figure 4D).

### 3.4 CCN3 and PTHLH are differentially expressed in A53T-PD1 iPS cell line

**FIGURE 5:**
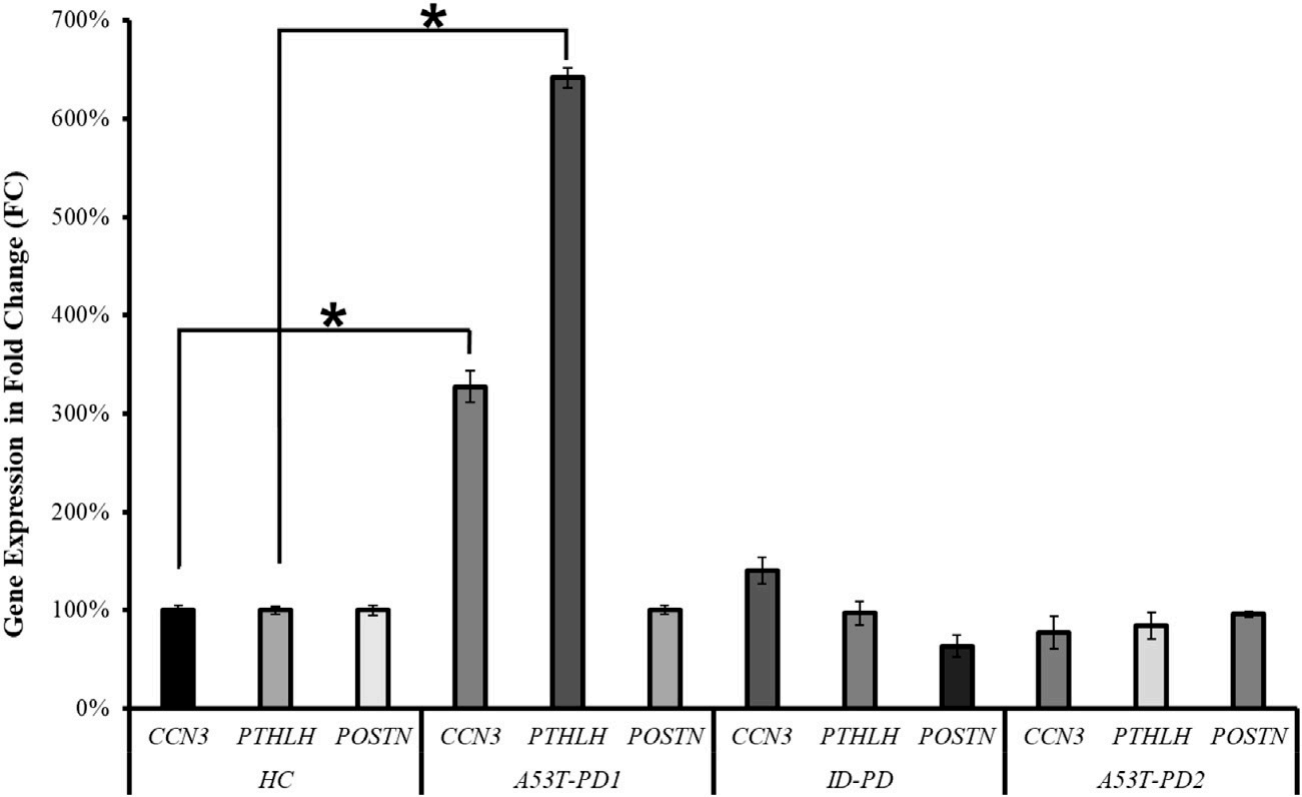
RT-qPCR validation of three candidate genes in various iPS cell lines. RT-qPCR analysis was conducted to validate the expression of the three candidate genes (*CCN3, PTHLH, POSTN*) across different iPS cell lines. The statistical significance was determined using the Mann–Whitney *U* test, with *p < 0.0001 indicating significance.

As a means of corroborating the RNA-seq analysis that identified *CCN3*, *PTHLH*, and *POSTN* as potential players in cellular stability and pluripotency maintenance, we quantified their expression levels across the four distinct iPS cell lines (HC, A53T-PD1, A53T-PD2, and ID-PD) using RT-qPCR. In terms of the genes under examination, the expression levels of *POSTN* in A53T-PD1 exhibited similarity to those observed in the control (HC) cell line, with insignificant downregulation observed in both the ID-PD and A53T-PD2 iPS cell lines. Conversely, the A53T-PD1 cell line exhibited significantly elevated expression levels of *CCN3* (227-fold increase, *p* < 0.0001) and *PTHLH* (542-fold increase, *p* < 0.0001) when contrasted with control iPS cell lines (Figure 5). These findings are consistent with our RNA-seq analysis, providing further support to our observation that additional transcriptional factors may play a role in bolstering the stability of iPS colonies and upholding their pluripotency.

It is imperative to note that differences, whether insignificant or significant, may exist between RNA-seq and RT-qPCR analyses of gene expression levels. Such variations can be attributed to several factors, including methodological disparities. RNA-seq provides a comprehensive, unbiased assessment of the entire transcriptome, detecting both known and novel transcripts, whereas RT-qPCR focuses on specific transcripts, offering a more targeted analysis. Additionally, differences in sensitivity and dynamic ranges are evident, with RNA-seq possessing a broader dynamic range capable of detecting low-abundance transcripts and capturing alternative splicing events, while qPCR, though highly sensitive, may have a more limited dynamic range compared to RNA-seq. Furthermore, normalization methods diverge, with RNA-seq utilizing techniques like TPM (transcripts per million) or FPKM (fragments per kilobase of transcript per million mapped reads) to account for library size and transcript length, while qPCR relies on reference genes for normalization, and the choice of reference genes can influence results. Lastly, considerations of gene isoforms and alternative splicing reveal that RNA-seq detects multiple isoforms and alternative splicing events, whereas primers designed for qPCR may target specific isoforms, potentially missing alternative splicing events (Marioni et al., 2008; Mortazavi et al., 2008; Bustin et al., 2009; Wang et al., 2009; Derveaux et al., 2010).

### 3.5 CCN3 involvement in cell apoptosis and FGFR2 signaling pathways

**FIGURE 6:**
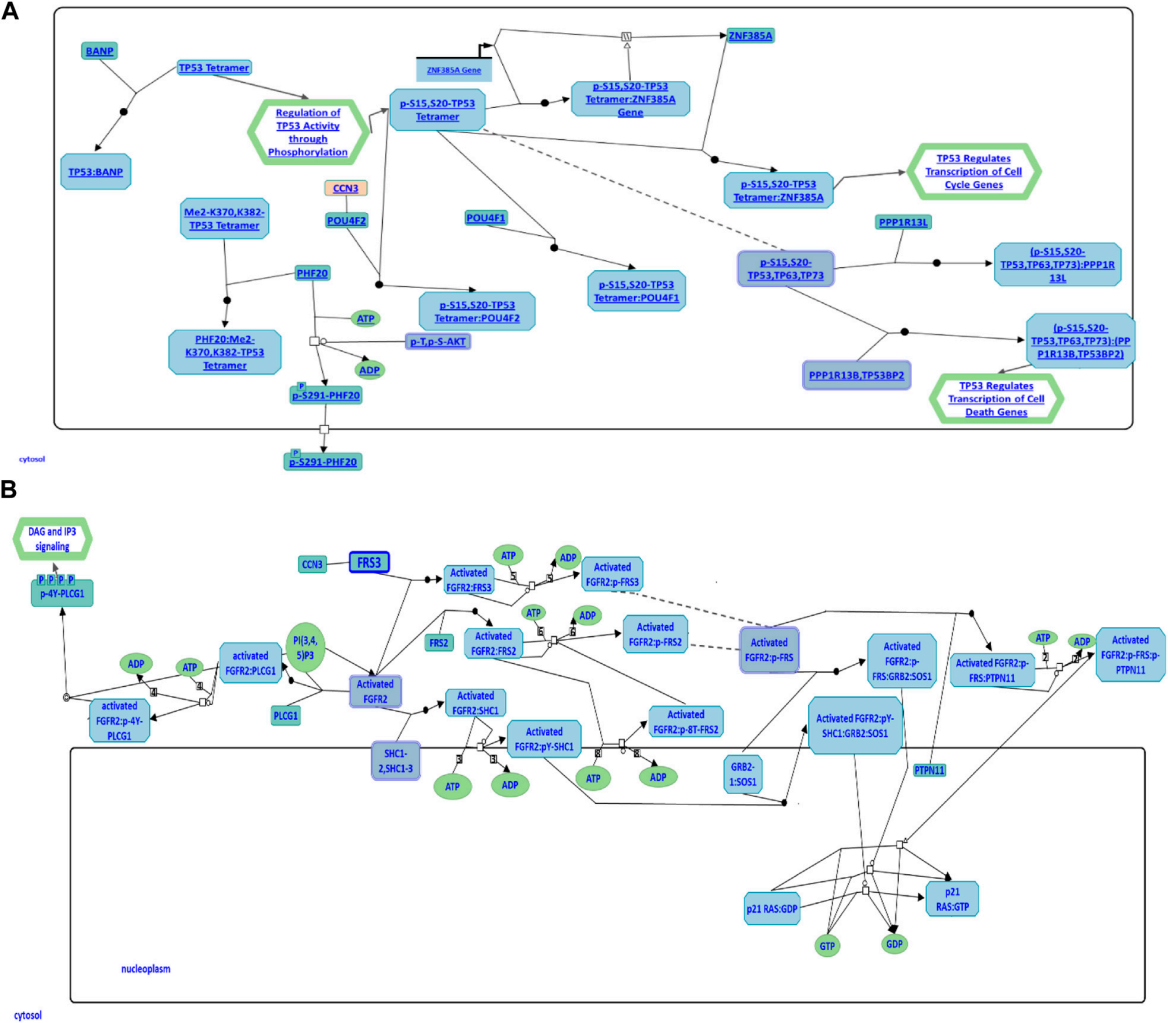
***CCN*** *3* involvement in cell apoptosis and *FGFR2* signaling pathways. **(A)** CCN3’s role in the p53 apoptosis pathway is illustrated. CCN3 interacts with the POU4F2 domain, which binds to TP53, regulating the transcription of pro-apoptotic genes. **(B)** CCN3’s involvement in the FGFR2 signaling pathway is represented. CCN3 interacts with FSR3, an adapter protein that transmits signals downstream from FGFR. Both diagrams have been obtained and adapted under the CC-BY 4.0 international license from Reactome.

To further explore *CCN3’*s role in the regulation of cellular apoptosis and proliferation, we conducted a comprehensive investigation using the Reactome Pathway Database. The data obtained from this database has revealed its participation in cellular apoptosis through an interaction with the POU4F2 domain, known to bind to TP53, thereby modulating the transcription of pro-apoptotic genes (Figure 6A). Inhibition of this pathway has been observed to augment iPSC reprogramming efficiency by fostering cell proliferation. Furthermore, *CCN3* has also been found to be a participant in the fibroblast growth factor receptor 2 (FGFR2) signaling pathway, where it interacts with fibroblast growth factor receptor substrate 3 (FSR3), an adapter protein responsible for transmitting signals downstream from FGFR (Figure 6B). Activation of this pathway has been demonstrated to play a crucial role in both the initiation and stabilization phases of reprogramming by promoting cellular proliferation.

### 3.6 PTHLH implication in G protein activation pathway

**FIGURE 7:**
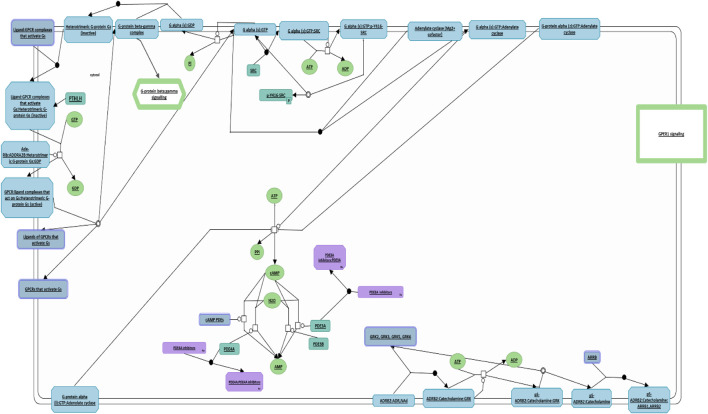
***PTHLH*-**mediated G protein activation pathway. This figure illustrates the pivotal role of *PTHLH* in G protein activation where it binds to G-protein-coupled receptor (GPCR), forming a liganded-GPCR complex. This diagram has been obtained and modified under the CC-BY 4.0 international license from Reactome.

Similarly, we conducted a comprehensive investigation utilizing the Reactome Pathway Database analysis to elucidate the cellular processes governed by *PTHLH*, leading to the discovery of its central role in G protein activation (Figure 7). *PTHLH’s* interaction with a G-protein-coupled receptor (GPCR) forms a liganded-GPCR complex, initiating the exchange of GDP for GTP within the G-protein alpha subunit (GNAS), ultimately resulting in G-protein activation. Once activated, the GNAS directly interacts with the proto-oncogene tyrosine kinase Src (SRC), leading to SRC autophosphorylation and subsequent activation. Functionally, the SRC kinase family plays a pivotal role in cellular growth and cancer. Additionally, the G-protein family, particularly the GNAS, is involved in the regulation of cellular apoptosis and the maintenance of pluripotency in ESCs.

## 4 Discussion

ESCs and iPSCs offer immense potential, not only in the field of regenerative medicine but also as valuable tools for investigating developmental processes, pathologies, and the assessment of novel drug interventions. However, the maintenance of genomic integrity and cellular stability presents a formidable challenge to sustaining the self-renewal and pluripotency of PSCs. Genomic instability can impair self-renewal pathways, activate the p53 apoptotic pathway, and promote PSC differentiation (Su et al., 2019). It is therefore imperative to safeguard the genomic integrity of PSCs and explore the genes that may be involved in this process, all while promoting robust growth to preserve their vital functions and self-renewal capability. Several factors, including culture conditions, can contribute to genomic instability in ESCs, and exposure to endogenous or exogenous genotoxic stresses can lead to cell apoptosis due to DNA damage (Martins-Taylor and Xu, 2012). It has been noted that ESCs have shown distinct mechanisms to shield themselves from DNA double-strand breaks, setting them apart from somatic cells (Nagaria et al., 2013).

In our previous study (Swaidan et al., 2020), iPS cell lines were successfully generated from a PD patient carrying the A53T mutation (A53T-PD1). Although the generated colonies number was limited, these colonies exhibited remarkable genomic resilience and prolonged viability compared to other iPS cell lines that were generated in parallel. This intriguing finding prompted us to consider additional factors influencing genomic integrity beyond age and disease mutation. Our hypothesis regarding the potential roles of certain molecules specific to A53T-PD1 cell lines, at both nuclear and cellular levels, in maintaining genomic and cellular stability were substantiated by transcriptomic analysis. This analysis revealed three differentially expressed genes in the A53T-PD1 cell line (*CCN3, POSTN,* and *PTHLH*), all of which are implicated in cellular stability, DNA repair mechanisms, and apoptosis inhibition.

Among these genes, *CCN3*, also known as *NOV*, exhibited significant upregulation in the A53T-PD1 iPS cell line compared to other cell lines (34.9-fold, FDR <0.001). *CCN3* belongs to the CCN family of matricellular proteins, known for its pivotal role in extracellular signaling and the maintenance of cellular viability (Kubota and Takigawa, 2013). Previous studies have highlighted the importance of *CCN3*, particularly its role in cellular adhesion and the regulation of integrin expression (Lafont et al., 2005; Lin et al., 2005; Vallacchi et al., 2008). These findings support our hypothesis that *CCN3* plays a crucial role in pluripotency circuits and their maintenance, particularly in cell adhesion receptor systems and integrin expression (Vitillo and Kimber, 2017; Yu et al., 2018). Moreover, the involvement of *CCN3* in regulating integrin expression not only underscores its significance in initiating and sustaining pluripotency but also implies its role in maintaining the compact morphology of pluripotent colonies, dependent on integrin activity (Yu et al., 2018). In addition, increased cellular proliferation, a hallmark of cells undergoing reprogramming, can be achieved by inhibiting *p53*, which is responsible for programmed cell death, and promoting *LIN28* expression, which regulates cell cycle genes like *CDK* genes. This mechanism contributes to the efficiency of reprogramming processes and the stability of hiPSCs (Hong et al., 2009; Kawamura et al., 2009; Hawkins et al., 2014).

**FIGURE 8:**
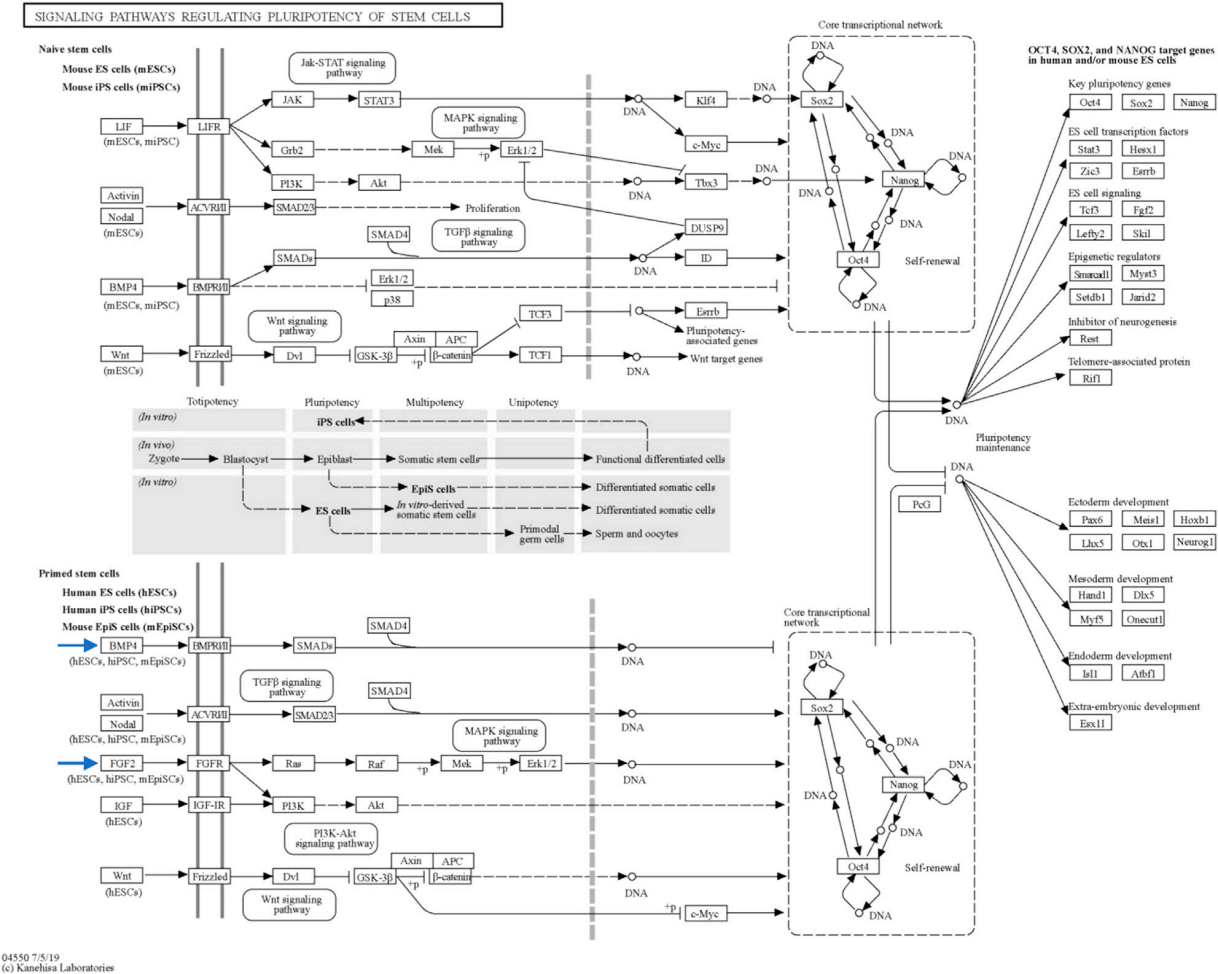
Key signaling pathways regulating stem cell pluripotency. This figure, sourced from the Kyoto Encyclopedia of Genes and Genomes (KEGG) database, provides an overview of the primary signaling pathways that govern the pluripotency of stem cells. The figure is interactive and can be explored in detail with links to pathway representations available at https://www.genome.jp/pathway/map04550. Of particular interest are the BMP4 and FGF2 pathways within primed stem cells (indicated by the blue arrows), which play significant roles in pluripotency regulation.

Through STRING analysis, we observed that *CCN3* is involved in promoting cell survival. Notably, it directly interacts with key players in the p53 pathway, including *TP53* and *CDKN1A*, which regulate cell apoptosis and survival. Reactome analysis further elucidated these interactions, revealing *CCN3’s* direct interaction with POU domain transcription factors that modulate p53 and regulate the transcription of pro-apoptotic genes (Figure 6A). When the P53 pathway is activated in stem cells, it initiates a cascade of events leading to cell cycle arrest, commitment to a differentiation pathway, and the formation of progenitor cells. P53 serves as a guardian of genomic stability, and its activation is pivotal for maintaining cellular integrity (Levine et al., 2016). Furthermore, *CCN3’s* interaction with *FGF2*, a key factor in the FGF2 pathway governing reprogramming initiation and stabilization, provides additional evidence of its role in improving reprogramming efficiency. *FGF2* accelerates cell proliferation during the initial phases of reprogramming, and it has been noted that hiPSCs generated in the presence of Activin/Nodal and *FGF2* ligands tend to stabilize in the primed state (Jiao et al., 2013; Hawkins et al., 2014). In addition, *FGF2* is known to influence the self-renewal and clonogenic capacity of pluripotent stem cells (Aprile et al., 2023). These findings highlight *CCN3’s* significance in orchestrating key signaling pathways that govern cellular fate and function. Its active participation in both the P53 and FGF2 pathways positions *CCN3* as a central player in the intricate network of molecular events that determine cell behavior, stability, and responsiveness to environmental cues. Our results were further substantiated by pluripotency pathways extracted from the Kyoto Encyclopedia of Genes and Genomes (KEGG) database, which highlighted the significance of the FGF2 pathway in governing reprogramming process in primed stem cells (Figure 8). Interestingly, the *CCN3* gene has been documented to collaborate with *FGF2* as a substrate, leading to enhanced cell proliferation and improved cell survival when they are combined (Lafont et al., 2005; van Roeyen et al., 2008). Reactome analysis also revealed *CCN3’s* direct interaction with FSR3, an adapter protein linking FGF receptor to downstream signaling pathways (Figure 6B).

Moreover, *CCN3* was found to enhance the expression of BMP-4, a protein critical for pluripotency initiation and maintenance through various mechanisms (Qi et al., 2004; Tan et al., 2012; Hawkins et al., 2014; Yu et al., 2020). KEGG database results highlighted the BMP-4 pathway’s involvement in governing reprogramming process in primed stem cells (Figure 8). Collectively, these findings strengthen our hypothesis regarding *CCN3’s* role in initiating and maintaining the reprogramming process, as well as its contribution to stabilizing and preserving reprogrammed cells from apoptosis.

The role of *CCN3* in cellular stability and integrity emerges as a complex and multifaceted process, as evidenced by the findings from various studies employing overexpression and knockdown approaches. A study focused on *CCN3* overexpression in bone marrow-derived stroma cell line (ST-2) cells revealed its inhibitory influence on BMP signaling, particularly preventing BMP-2 from phosphorylating Smad1/5/8, ultimately leading to the inhibition of osteoblastogenesis. This implies that *CCN3* may play a crucial role in regulating bone homeostasis by modulating BMP signaling, a pathway fundamental to osteoblastic differentiation (Zuo et al., 2010), which in turn indicates the stability of ST-2 cells in its undifferentiated form. Moreover, *CCN3’s* involvement in the regulation of primitive hematopoietic stem cells adds another layer to its significance in cellular homeostasis. Studies employing *CCN3* knockdown or forced expression in CD34^+^ cells have shown the necessity of *CCN3* for the functional self-renewal of hematopoietic stem cells (Zuo et al., 2010).In a different investigation, *CCN3* levels were modified by employing adenoviruses that either overexpressed *CCN3* (Ad-CCN3) or interfered with *CCN3* (Ad-siCCN3) in mouse embryonic fibroblasts (MEFs). This study aimed to examine the impacts and mechanisms of *CCN3* on the process of osteoblastic differentiation. The findings indicated a notable hindrance of osteogenic differentiation in MEFs with higher *CCN3* expression, primarily attributed to the suppression of BMP signaling and the reciprocal inhibition with DLL1(Su et al., 2018). Mechanistic analyses further uncovered *CCN3’s* suppressive effects on BMP/Smad and BMP/MAPK signaling pathways, along with its mutual inhibition with *DLL1*, a prominent membrane protein ligand associated with the Notch signaling pathway. Significantly, Hey1, a target gene shared by both BMP and Notch signaling pathways, emerged as a pivotal factor, partially counteracting the inhibitory impact of *CCN3* on osteoblastic differentiation (Su et al., 2018). These regulatory roles of *CCN3* in both bone formation and hematopoiesis underscores its vitality in maintaining cellular stability within distinct cellular contexts.

Another gene exclusively upregulated in the A53T-PD1 iPS cell line compared to other cell lines was *PTHLH* (59-fold, FDR <0.001). STRING analysis unveiled an unknown interaction between *PTHLH* and *CDK2*, which plays a role in the cell cycle and *TP53* inhibition. Previous research has highlighted *PTHLH* as a crucial molecular regulator of cellular development and survival (Guo et al., 2012). Additionally, in Pthlh-depleted embryos at the morula stage, the expression of pluripotency-related genes *NANOG* and *POU5F1* was markedly diminished (Min et al., 2022). While there is no direct evidence suggesting *PTHLH’s* involvement in histone acetylation during early embryonic development before implantation, previous research has indicated its direct influence on histone deacetylase 4 (HDAC4) (Kozhemyakina et al., 2009; Correa et al., 2010). Moreover, *PTHLH* exerts its influence through a complex network of signaling pathways involving ERK/MAPK pathways (Datta et al., 2005). ERK depletion has been associated with dysregulated pluripotency gene expression, decreased proliferation rates, G1 cell cycle arrest, increased apoptosis, faster telomere shortening, and impaired genomic stability (Chen et al., 2015). This data correlates well our data that *PTHLH* may play a role in increasing the cell survival and stability.

Reactome analysis confirmed *PTHLH’s* role in intracellular signaling, through its binding to GPCR leading to the activation of GNAS (Figure 7). G-proteins are essential regulators of cellular apoptotic cascades, with GNAS being linked to both proapoptotic and anti-apoptotic pathways (Melien, 2007; Yanamadala et al., 2009). GNAS overexpression has been shown to inhibit hydrogen peroxide-induced apoptosis by downregulating Bcl-xl (Zhao et al., 2006). Furthermore, endogenous GNAS activation through agonists has inhibited apoptosis mediated by cAMP production and PKA activation, leading to the inhibition of pro-apoptotic transcription factors such as AP-1, NF-κB, and NFAT (Choi et al., 2009). Interestingly, the Gs-alpha pathway has been proposed to regulate pluripotency in ESCs. Activation of Gs-alpha and amplification of cAMP levels have been shown to promote Oct4 expression, stimulating ESC self-renewal and pluripotency (Faherty et al., 2007; Layden et al., 2010). Therefore, we hypothesize that *PTHLH* may exert indirect influences on the expression of pluripotency-related genes, leading to more cellular stability, although the precise underlying mechanism warrants further investigation.

*PTHLH* has shown to be an upstream activator of GNAS which directly interacts with SRC, leading to SRC autophosphorylation and subsequent activation. SRC plays a diverse role in iPSCs, influencing key cellular processes such as survival, proliferation, and cell fate decisions. Its signaling pathways support iPSC self-renewal, prevent apoptosis, maintain stability, and impact commitment to specific lineages (Lu et al., 2007; Shoni et al., 2014; Zhang et al., 2014; Chaudhari et al., 2019). SRC also modulates pluripotency-associated pathways, including Wnt/β-catenin (Min et al., 2022), which is crucial for maintaining stem cell identity. During cellular reprogramming, SRC activation facilitates the transition to a pluripotent state, contributing to the erasure of cell identity (Ma et al., 2013; González and Huangfu, 2016). Additionally, SRC is associated with cellular senescence, potentially regulating senescence during reprogramming (Anerillas et al., 2022). The context-dependent nature of SRC in iPSCs, influenced by cellular context, reprogramming stage, and interactions with other pathways, underscores the dynamic complexity of its functions. Altogether, emphasize the important role of *PTHLH* in maintaining iPSC stability and integrity. Indeed, the downregulation of *PTHLH* emerged as a critical factor influencing cellular stability and integrity, particularly evident in a *MYCN*-amplified, TP53-mutated neuroblastoma cell line. The reduction of *PTHLH* levels led to a consequential decrease in *MYCN* expression. This event triggered a cascade of effects, including cell cycle arrest, induction of senescence, and impairment of migration and invasion capabilities within the neuroblastoma cell line (García et al., 2019). These findings highlight the intricate role of *PTHLH* in orchestrating key cellular processes and maintaining the equilibrium necessary for stability and integrity.

*POSTN* exhibited the highest upregulation, increasing by 70-fold (FDR <0.001), in A53T-PD1 iPS cell line compared to other iPS cell lines (Figure 3B). STRING analysis predicted that *POSTN* interacts with extracellular matrix components, including collagen-related proteins, Decorin and Tenascin C. Our findings align with previous research indicating that *POSTN* is highly expressed in a mouse ES cell line treated with Mek inhibitor PD0325901, in contrast to other untreated cell lines (Chen et al., 2015). PD is a pharmacological agent that inhibits the Mek/Erk signaling pathway, promoting self-renewal and pluripotency in mESCs (Chen et al., 2015). Additionally, *POSTN* has been associated with the augmentation of the Wnt signaling pathway in mouse breast cancer stem cells (Wang et al., 2013). This suggests a potential role for *POSTN* in maintaining self-renewal and pluripotency through the activation of the Wnt signaling pathway, which is essential for preserving naïve pluripotency and epigenetic stability in mESCs (de Jaime-Soguero et al., 2018; Theka et al., 2019).

Previous studies elucidate the intricate and context-dependent role of *POSTN* in cellular stability and integrity across various biological systems. In renal cell carcinoma (RCC), *POSTN* emerges as a key regulator, where its knockdown significantly suppresses epithelial-mesenchymal transition (EMT) through the IKL/AKT/mTOR pathway. Conversely, overexpression of *POSTN* facilitates EMT (Jia et al., 2021), indicating the crucial role of *POSTN* in cell stability and integrity. Moreover, overexpression of *POSTN* in the context of osteoblasts acts as a protective factor by impeding melatonin-induced apoptosis; hence, increase cell stability and integrity. This protective effect is achieved by inhibiting the eIF2α-ATF4 pathway through the suppression of the protein kinase R-like endoplasmic reticulum kinase (PERK) pathway (Zhu et al., 2021). These roles of *POSTN* highlights its versatile functions in maintaining cellular homeostasis. Furthermore, using mesenchymal stem cells (MSCs), *POSTN* plays a crucial role in the mineralization of the extracellular matrix (ECM) and supports tendon formation when overexpressed. This emphasizes *POSTN’s* involvement in ECM dynamics, which is essential for tissue development and integrity (Zhu et al., 2021).

In conclusion, our study sheds light on the potential roles of *CCN3, PTHLH*, and *POSTN* in maintaining genomic and cellular stability while preserving pluripotency in iPSCs. These findings warrant further investigations to unravel the precise mechanisms through which these genes contribute to these processes.

## 5 Conclusion

In summary, we identified three genes that were highly expressed in A53T-PD1 iPS cell line, from which robust and stable colonies were generated. The implications of these identified genes extend to potential selective interactions, which are vital for upholding the genomic integrity and maintaining pluripotency in both ESCs and iPSCs. Their functions may encompass activating DNA repair mechanisms, evading apoptosis through TP53 phosphorylation, and triggering signaling pathways that are crucial for preserving the pluripotent state of ESCs. To substantiate this conceptual framework and gain a deeper understanding of the underlying mechanisms, it becomes imperative to conduct thorough functional investigations. These endeavors hold the promise of unraveling crucial insights into the precise roles of these genes in ensuring genomic stability while upholding the self-renewal and pluripotency of the generated iPSCs.

## Data Availability

The raw data supporting the conclusion of this article will be made available by the authors, without undue reservation.
